# CD-REST: a system for extracting chemical-induced disease relation in literature

**DOI:** 10.1093/database/baw036

**Published:** 2016-03-25

**Authors:** Jun Xu, Yonghui Wu, Yaoyun Zhang, Jingqi Wang, Hee-Jin Lee, Hua Xu

**Affiliations:** 1 School of Biomedical Informatics, The University of Texas Health Science Center at Houston, Houston, TX 77030, USA

## Abstract

Mining chemical-induced disease relations embedded in the vast biomedical literature could facilitate a wide range of computational biomedical applications, such as pharmacovigilance. The BioCreative V organized a Chemical Disease Relation (CDR) Track regarding chemical-induced disease relation extraction from biomedical literature in 2015. We participated in all subtasks of this challenge. In this article, we present our participation system Chemical Disease Relation Extraction SysTem (CD-REST), an end-to-end system for extracting chemical-induced disease relations in biomedical literature. CD-REST consists of two main components: (1) a chemical and disease named entity recognition and normalization module, which employs the Conditional Random Fields algorithm for entity recognition and a Vector Space Model-based approach for normalization; and (2) a relation extraction module that classifies both sentence-level and document-level candidate drug–disease pairs by support vector machines. Our system achieved the best performance on the chemical-induced disease relation extraction subtask in the BioCreative V CDR Track, demonstrating the effectiveness of our proposed machine learning-based approaches for automatic extraction of chemical-induced disease relations in biomedical literature. The CD-REST system provides web services using HTTP POST request. The web services can be accessed from http://clinicalnlptool.com/cdr. The online CD-REST demonstration system is available at http://clinicalnlptool.com/cdr/cdr.html.

**Database URL:** http://clinicalnlptool.com/cdr; http://clinicalnlptool.com/cdr/cdr.html

## Introduction

Over the past decades, extensive biomedical studies have been conducted to assess the relations between chemicals and diseases, which resulted in a huge volume of literature regarding complex chemical–disease relations (e.g. treatment or adverse events). Significant efforts have been spent on building comprehensive databases containing relations between chemicals and diseases from literature. As an example, the Comparative Toxicogenomics Database (CTD) (1) contains chemical–disease associations that are manually extracted from the biomedical literature by biocurators. Although manual review of literature helps generate accurate knowledge, it is very time-consuming, given the rapid growth of published literature. Therefore, natural language processing (NLP) methods that could automatically detect chemical and disease concepts, as well as their relations from biomedical literature have shown great potential in terms of facilitating biomedical curation processes (2–4). Automated extraction of chemical and disease relations from literature requires two steps: 1) named entity recognition (NER), to identify chemical and disease entities from narrative text; and 2) relation extraction, to determine the relations between any pair of chemical and disease entities in one document.

Many attempts have been made for chemical and disease NER, by using different approaches. For example, many NER systems are rule-based, relying on existing biomedical databases/dictionaries. Among them, cTAKES (5) and MetaMap (6) are two widely used systems for extracting various types of entities including chemicals/drugs and diseases and linking them to concepts in the Unified Medical Language System (UMLS) (7), for clinical narratives and biomedical literature respectively. LeadMine (8) uses grammars and dictionaries to recognize chemical entities. In addition, many high-performance biomedical NER systems were developed based on annotated corpora using machine learning algorithms. Jiang *et al.* (9) implemented a machine learning-based system to extract clinical entities, including medical problems, tests and treatments from narrative clinical notes. Leaman *et al.* (10) developed a high-performance chemical NER and normalization system—tmChem, which was the best performing system in the BioCreative IV CHEMDNER task. Researchers have also proposed hybrid approaches for NER, such as the ChemSpot (11) system for chemical and the UTH-CCB (12) system for disease recognition. The successes of these hybrid systems indicate that the traditional machine-learning-based biomedical NER systems can be further improved by integrating with rules.

Relation extraction from biomedical literature is another important task of NLP (13). It has received great attention and many different approaches have been developed (14). Common relation extraction methods initiated in the general domain, such as co-occurrence analysis, rule-based methods, and machine-learning-based methods, have been applied to chemical–disease relation extraction. Chen *et al.* (15) conducted co-occurrence analysis to rank the associations between eight diseases and relevant drugs. Mao *et al.* (16) also used co-occurrence analysis to identify aromatase inhibitors-related adverse drug events in health social media. The rule-based approaches often relied on manually developed rules based on syntactic and semantic parsing. Khoo *et al.* (17) explored manually annotated graphical patterns to extract causal relations in the MEDLINE abstracts by using syntactic parse trees. The MeTAE system extracted medical relations based on semi-automatically constructed linguistic rules (18). Instead of manually constructing rules, Xu and Wang (19) designed a system to learn drug–side-effect-specific syntactic patterns from the parse trees using known drug–side-effect associations as a clue. Then, they used the learned patterns to extract additional drug–side-effect pairs from biomedical literature. Researchers have also applied machine-learning approaches to extract chemical–disease relations. For example, Rosario and Hearst (20) compared graphical models and neural networks on the identification of the semantic relations between diseases and treatments using lexical, syntactic and semantic features. Gurulingappa *et al.* (21) trained support vector machines to extract potential adverse drug event relations from the MEDLINE case reports.

However, most of the previous studies on chemical–disease relation extraction focused on either the entity recognition or the relation extraction—few of them provide an end-to-end solution. Moreover, the identified entities were not normalized to standard terminologies. In 2015, the BioCreative V introduced a shared task on Chemical Disease Relation (CDR) extraction, which consists of two subtasks: 1) Disease NER and Normalization (DNER); 2) Chemical-induced Diseases Relation Extraction (CID), which is to extract all chemical-induced disease pairs asserted in one abstract (22). It requires all participants to identify chemical and disease entities and then the chemical-induced disease relations.

In this article, we present the Chemical Disease Relation Extraction SysTem (CD-REST) built for the BioCreative V CDR Track. CD-REST consists of two modules: 1) an entity recognition and normalization module that recognizes chemicals and diseases using Conditional Random Fields (CRFs) (23) and normalizes them into Medical Subject Headings concept identifiers (MeSH ID) using a vector space model (VSM)-based approach, and 2) a relation extraction module that extracts chemical-induced disease relations from both the sentence and document levels using support vector machine-based classifiers. CD-REST achieved the best performance on the CID task in the BioCreative V CDR Track, demonstrating the effectiveness of our proposed machine-learning-based approaches for automatic extraction of chemical-induced disease relations from biomedical literature.

## Materials and methods

### Datasets

**Figure 1.**
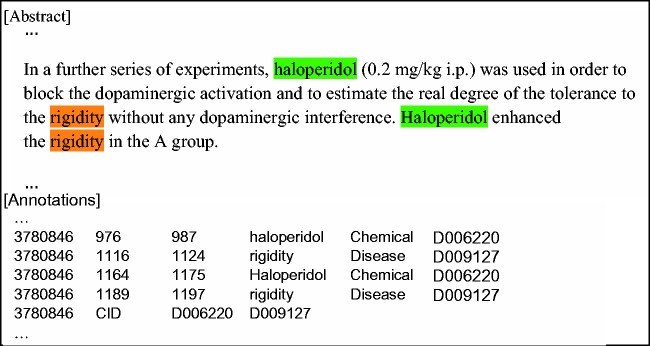
A sample from the CDR corpus with the annotations of mentions, corresponding normalized MeSH IDs for both chemical and disease entities and normalized chemical-induced disease relation conveyed in the abstract.

The CDR Track organizers developed a corpus (the CDR corpus) for NER and chemical–disease relation extraction using a set of PubMed abstracts. This corpus consists of 1500 abstracts with 4409 annotated chemicals, 5818 diseases and 3116 chemical–disease interactions (24). As illustrated in Figure 1, the annotators manually annotated entity text spans and then normalized the entities to MeSH ID. The relations between chemicals and diseases were annotated at the document level (without indicating the specific sentence(s) that contributed to the relations). In the CDR Track, the corpus was divided into a training set (500 abstracts), a development set (500 abstracts) and a test set (500 abstracts).

### System description

**Figure 2.**
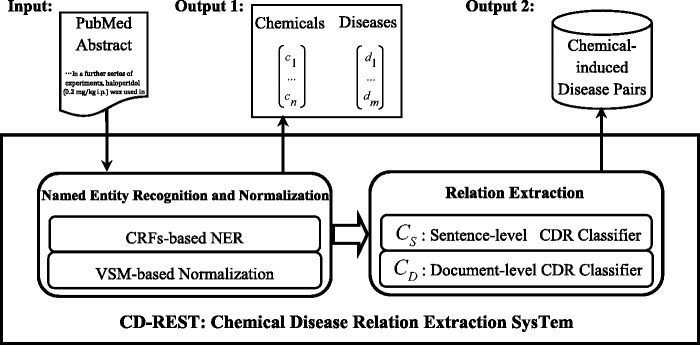
An overview of CD-REST.

The CD-REST system that we proposed is an end-to-end approach to extract chemical-induced disease relations from biomedical literature. Figure 2 shows the workflow of the CD-REST system. We employed CRF-based NER approaches for chemical and disease entities, by making use of different types of features including distributed word representation features learned from unannotated corpus. We adopted a VSM-based approach to normalizing recognized entities into MESH IDs by calculating the similarity between the target entity and candidate MeSH concepts. Then, we trained two classifiers to extract chemical–disease relations at sentence and document levels respectively and combined their outputs to generate final relation pairs. We describe the details in the following sections.

### NER and normalization

#### Entity representation

Both disease and chemical recognition are typical NER tasks. We transformed the annotated data into the BIO format, in which “B-D” and “I-D” were used to denote the begin- and continuation- of the disease entity, respectively. Similarly, “B-C” and “I-C” tags were used for the chemical entity. “O” was used for any tokens outside of any entities.

#### Machine-learning algorithm

We employed CRFs for both chemical and disease NER. The implementation in the CRFsuite package (http://www.chokkan.org/software/crfsuite/) was used in this study.

#### Features

We systematically investigated different types of features for chemical and disease NER, including: 1) Word-level features: Bag-of-word, Part of Speech (PoS) tags, orthographic information, such as case patterns, char n-gram, prefixes and suffixes of words; 2) Dictionary lookup features: We developed a dictionary-based semantic tagger by leveraging existing vocabularies and corresponding semantic tags (e.g. disorder, problem, drug, etc.) from UMLS; 3) Contextual features: Bi- and tri-grams of tokens, including word, word stem, PoS and semantic tags extracted by our semantic tagger; 4) Chemical/disease-related features: We adopted the features representing characteristics specific to chemicals from tmChem (10). We also defined several binary features for diseases, including suffixes (e.g. “-algia”, “-emia”, etc.) and prefixes (e.g. “ab-”, “hemo-”, etc.); and 5) Distributed word representation features: In this study, we explored the deep neural network-based word embeddings. We developed a deep neural network (25) to train word embeddings from all PubMed abstracts published in 2013.

#### Named entity normalization

We adopted a previously developed VSM-based encoding module (12) to find the correct MeSH ID for a given entity. This encoding module was originally developed to normalize clinical entities into UMLS concept identifiers using a term to UMLS CUI index. In this study, we re-built the index using MeSH. We calculated the cosine similarity scores between a target entity and all candidate concepts in MeSH and returned the MeSH ID which has the highest similarity score. If the target entity hits multiple MeSH IDs with the same score, we randomly select one. When there was no MeSH ID matching the target entity, the normalization module will assign “-1” as a pseudo ID as required by the challenge guidelines.

### Chemical-induced disease relation extraction

We treated the chemical-induced disease relation extraction task as a binary classification problem. Although the CDR corpus only provided document-level annotations, we separated relations at the sentence level and the document level, by developing a sentence-level classifier (*C_s_*) and a document-level classifier (*C_D_*) to identify the CDR pairs using the evidences from a sentence or an abstract.

#### Sentence-level classifier

The *C_s_* classifier utilized intra-sentence text features incorporating with domain knowledge to identify chemical-induced disease pairs located in the same sentence. As Tables 1 and 2 show, we systematically investigated three different groups of features:

**Table 1.** The entity and context information features used for the sentence-level classifier *C*_S_ and the document-level classifier *C*_D_

**#**	**Name**	**Gloss**	***C*_S_**	***C*_D_**
*Entity information*				
1	Entity mention	Bag of words & bigrams of the entity mentions	√	√
2	Chemical first	Is chemical the first entity in the sentence	√	
3	MeSH Ids	The corresponding MeSH IDs of each entity	√	√
4	Core chemical	Whether target chemical is a core chemical	√	√
*Context information*				
5	Before	Bag of words & bigrams before the entities	√	
6	Between	Bag of words & bigrams between the entities	√	
7	After	Bag of words & bigrams after the entities	√	
8	Same sentence	Whether the <c,d>pair locates in the same sentence		√
9	Adjacent sentences	Whether the <c,d>pair locates in adjacent sentences		√
10	More than two sentences	Whether the <c,d>pair crosses more than two sentences		√
11	Match *terms*(*i*)	Whether the words between the entities contains any term in *terms*(*i*) that indicated the **i**nduced relation, such as “caused”, “induced” etc.	√	√
12	Match *terms*(*h*)	Whether the sentence contains *d* has any term in *terms*(*h*)that indicated the **h**older of *d*, e.g. “patient”, “groups” and “rats” etc.	√	√(if feature 8 or 9 is true)

**Table 2.** Features extracted by incorporating knowledge bases

**#**	**Name**	**Gloss**
*MeSH features*		
1	Categories of *d*	All direct or indirect hypernyms of *d*
2	Categories of *c*	All direct or indirect hypernyms of *c*
3	Has a specific disease	Whether the document has a more specific disease
4	Has a general disease	Whether the document has a more general disease
*MEDI features*		
5	r(<c,d>)	Relation of <c,d>in MEDI: *null or treatment*
6	r(<c,d>)	Relation of <c,d>in MEDI’s high precision subset
*SIDER features*		
7	r(<c,d>)	Relation of <c,d>in SIDER: *null, treatment or aderver-drug-reaction*
8	r(<c,d>)	Relation of <c,d>in SIDER subset confirmed by FDA Adverse Event Reporting System (26)
9	isADR(d)	Whether *d* is an adverse drug event in SIDER
*CTD features*		
10	r(<c,d>)	Relation of <c,d>in CTD: *null, inferred-association, therapeutic* or *marker/mechanism*
11	isInduced(d)	Whether *d* has a *marker/mechanism* association with any chemicals in CTD

These features were used for both *C*_S_ and *C*_D_ classifiers

*Context Information*: uni- and bi-gram of words before, between and after the target chemical and disease entities. Also, the presence of trigger words (e.g. induce) in the sentence was also used as features.

*Entity Information*: mentions and normalized values of the target chemical and disease entities. In addition, we defined a binary feature called “core chemical.” If a chemical entity occurs in the title or it is the most frequently mentioned chemical in the abstract, we define it as a “core chemical.”

*Information from domain knowledge*: the existing domain knowledge of the target chemicals and diseases. We explored four different knowledge bases: MeSH, CTD, MEDication Indication Resource (MEDI) (27) and Side Effect Resource (SIDER) (28). We converted all terms (chemicals/drugs and disease/ADRs) in the MEDI and SIDER into MeSH ID using UMLS. As shown in Table 2, we extracted all relations of the chemical–disease pair in the CTD, MEDI and SIDER as features. Chemicals or diseases from the same category are more likely to have similar biological properties. Thus, we extracted category-related features for each entity from its MeSH hierarchical tree structures, which were represented by several MeSH Tree Numbers (TN). Take the disease “retrograde amnesia” as an instance, all direct and indirect hypernyms, i.e. “C10”, “C10.597”, “C10.597.606” and “C10.597.606.525”, were extracted as categories by parsing its MeSH TN “C10.597.606.525.100”. In addition, based on the MeSH Tree Structures, we also re-visited the document to query whether the document had a more specific (hyponym) or general (hypernym) disease than the target disease. For example, “retrograde amnesia (C10.597.606.525.100.150)” is more specific than “amnesia (C10.597.606.525.100)”. Therefore, we were able to extract two binary features for each disease to denoting whether the source document has diseases more specific or general than the target disease.

#### Document-level classifier

The *C_D_* classifier utilized document-level information as well as domain knowledge to classify the relations between chemicals and diseases at the document level. The *C_D_* used above three groups of features from the *C_s_*. As shown in Table 1, compared to *C_s_*, *C_D_* also used the co-occurrence information of the target chemical and disease entities, but did not use the uni- and bi-gram features as in the *C_s_*.

#### Machine learning

For both sentence- and document-level relation classification, we employed SVMs algorithm and used the LIBSVM (29) package for SVMs implementation.

#### Training corpus generation

The training of the document-level classifier was straightforward as the relations were annotated at the document level in the gold standard. However, we needed to construct sentence-level annotations to train the sentence-level classifier. We extracted all sentences that had at least one chemical–disease pair, denoted as <c,d>, and generated the sentence-level annotations according to the document-level annotations by following a simple rule: a sentence-level relation pair <c,d> would be annotated as “*true*” if and only if the <c,d> pair is in the document-level annotations; otherwise, the <*c, d*> pair would be annotated as “*false*”.

### Experiments and evaluation

We developed our machine-learning models using the training set and optimized the parameters using the development set. Then we combined the training and the development datasets to build the final models.

#### NER and normalization

We tried two different approaches: (1) NER-S: trained two separate CRFs models, one for disease entities and the other for chemical entities, and (2) NER-U: trained a unified CRFs model for both disease and chemical entities. In the NER-S approach, additional external corpora were also investigated. We used the BioCreative IV CHEMDNER corpus (30) for chemical NER and the NCBI Disease Corpus (31) for disease NER.

#### Relation extraction

The CID task in the BioCreative V CDR Track was designed to extract CDRs in an end-to-end setting, in which predicted chemicals and diseases were provided as inputs to the relation extraction system. To better understand the performance of the relation extraction system, we also evaluated and reported the performance of the CDR extraction system using the gold-standard chemical and disease entities as the inputs. Three different strategies for generating chemical–disease pairs were used: (1) $C_{\text{S}}$, which applies $C_{\text{S}}$ for those <c,d> pairs located in the same sentences only; (2) $C_{\text{D}}$, which applies $C_{\text{D}}$ for all <c,d> pairs in the same document; and (3) *C_S_* + *C_D_*, a combination strategy of *C_S_* and *C_D_* in which the union set of the two classifiers’ predictions were used as our system’s predictions. Moreover, we evaluated the contribution of features from different domain knowledge bases.

#### Evaluation metrics

The evaluation metrics of the CDR track include *F*-score (F), precision (P) and recall (R). For DNER, the evaluation scores were calculated based on tuples of the document ID and the disease concept ID. In addition to the concept-level evaluation scores, we further reported P, R and F on the mention-level using exact span matching. This evaluation setting was also used for CNER. For the CID task, the evaluation scores were calculated based on 3-tuple composed of document ID, chemical and disease concept ID. Please refer to the task description (22) for more details.

## Results

Table 3 shows the performance of the CD-REST on chemical and disease NER and normalization task. The NER-S approach, which trained individual models for CNER and DNER, outperformed the NER-U approach that combined the chemical and disease entities recognition in one model. The best performance of DNER was achieved by the NER-S approach that used the CDR corpus only for model training. The best performance for CNER was achieved by the NER-S approach that used both the CDR corpus and the BioCreative IV CHEMDNER corpus for model training.

**Table 3.** Performance of the CD-REST in the CNER and DNER tasks on the test set with different approaches

**Task**	**# Run**	**Approach**	**Training dataset**	**Concept-level**			**Mention-level**		
				**P**	**R**	**F**	**P**	**R**	**F**
CNER	1	U	V	0.8850	0.9115	0.8980	0.9278	0.8858	0.9063
	2	S	V	0.8941	0.9112	0.9027	0.9339	0.8819	**0.9072**
	3	S	V+IV	0.9010	0.9199	**0.9103**	0.9376	0.8698	0.9024
DNER	1	U	V	0.8254	0.8395	0.8324	0.8648	0.8230	0.8434
	2*	S	V	0.8312	0.8395	**0.8353**	0.8689	0.8210	**0.8443**
	3	S	V+N	0.8158	0.8355	0.8255	0.8636	0.8232	0.8429

U: the NER-U approach; S: the NER-S approach; V: the BioCreative V CDR Corpus; IV: the BioCreative IV CHEMDNER Corpus; N: the NCBI Disease Corpus. * was the best run the CD-REST achieved on DNER task in the CDR challenge. DNER Run #3 was not submitted to the challenge. Where applicable, the best performance in each category is highlighted in bold.

Table 4 shows the performance of different approaches on the CID task in the end-to-end setting and the gold-standard setting. The *C_S _*_+_*_ _C_D_* approach outperformed individual classifiers (*C_S_* or *C_D_*), achieving the highest *F*-scores of 0.5853 in the end-to-end setting and 0.6716 when gold-standard chemical and disease entities were used.

**Table 4.** The performance of the CD-REST in the CID task using the end-to-end setting (CNER #1, DNER #1) and the gold-standard setting on the test set with different approaches. Where applicable, the best performance in each category is highlighted in bold.

**Approach**	**End-to-end**			**Gold-standard**		
	**P**	**R**	**F**	**P**	**R**	**F**
*C*_S_	**0.6424**	0.4381	0.5209	**0.6763**	0.5487	0.6059
*C*_D_	0.6412	0.5047	0.5648	0.6836	0.6182	0.6493
*C*_S_ + *C*_D_	0.6186	**0.5553**	**0.5853**	0.6580	**0.6857**	**0.6716**

Table 5 shows the performance of the CD-REST on the test set with features from different knowledge base features, based on the best performing strategy (*C_S _*+*_ _C_D_*). All features from knowledge bases improved the system’s performance. It is also not surprising that CTD improved the performance most, comparing with other knowledge bases, as CTD is the knowledge base for chemical-induced diseases.

**Table 5.** Results of the CD-REST with + approach on the test set using the end-to-end setting (CNER Run #1, DNER Run #1) and the gold-standard setting, when different sets of knowledge base features were used. The best results are highlighted in bold.

**Feature set**	**End-to-end**			**Gold-standard**		
	**P**	**R**	**F**	**P**	**R**	**F**
Entity + Context	0.5160	0.3640	0.4268	0.5960	0.4400	0.5073
Entity + Context + MeSH	0.5155	0.4222	0.4641	0.5842	0.5140	0.5469
Entity + Context + MeSH + MEDI	0.5206	0.4278	0.4696	0.5953	0.5244	0.5576
Entity + Context + MeSH + MEDI + SIDER	0.5308	0.4372	0.4794	0.6086	0.5310	0.5671
Entity + Context + MeSH + MEDI + SIDER + CTD	**0.6186**	**0.5553**	**0.5853**	**0.6580**	**0.6857**	**0.6716**

Table 6 shows performance of the CD-REST on the CID task using different combinations of CNER and DNER. Among all the combinations, the Run #1 achieved the highest *F*1-score of 0.5853. To our surprise, the Run #3, which combined the best CNER module (CNER #2) and the best DNER module (DNER #2), was outperformed by Run #1. Therefore, we further examined the two runs by calculating the “relation coverage”—defined as the number of gold standard relations covered by the predicted entities. A relation is labelled as covered if both the chemical entity and the disease entity were identified. We compared the relation coverage of the two runs based on the gold standard and found that the Run #1 covered 10 more relations than the Run #3, suggesting that the Run #1 could capture more in-relation entities than the Run #3.

**Table 6.** The performance of the CD-REST with *C*_S_ + *C*_D_ approach on the CID task using different combinations of CNER and DNER. Where applicable, the best performance in each category is highlighted in bold.

**#**	**# CNER Run**	**# DNER Run**	**P**	**R**	**F**
1	1	1	0.6186	**0.5553**	**0.5853**
2	2	2	0.6216	0.5516	0.5845
3	3	2	**0.6255**	0.5422	0.5809
4	2	3	0.6193	0.5525	0.5840
5	3	3	0.6231	0.5413	0.5793

During the challenge, we developed a rule-based post-processing module, which improved the performance on the development corpus. However, adding the post-processing module actually hurt the performance. Our best submission in the challenge (using strategy in CID Run #1 with the post-processing module) achieved the highest *F*-score (0.5703) among all teams, which is lower than the score reported in this article.

We examined the efficiency of CD-REST system using a computer with 32 GB RAM and a 3.7 GHz 4-core processor. It took about 450 s to process the whole test set for relation extraction. The average processing time for one abstract was <1 s. However, the web service took more time since it only processed one document per request (22).

## Discussion

In this study, we developed CD-REST, an end-to-end system to extract chemical-induced disease relations from biomedical literature by incorporating domain knowledge into machine-learning models. Our system achieved the best performance among 18 participating teams and 46 submitted runs in the challenge of the BioCreative V CDR Track. Our results demonstrated the feasibility of incorporating domain knowledge into machine-learning-based approaches for CDR extraction.

### System performance comparison and analysis

#### NER-S vs. NER-U

As shown in Table 3, NER-S, which trained individual classifiers for chemicals and disease, respectively, outperformed the NER-U approach, which combines chemical and disease entities into one model. We noticed that the NER-S approach always achieved a higher precision while maintaining a comparable recall. In general, a unified NER model built for all entities will benefit from the dependencies among different types of entities. However, the unified model performed worse in this study, probably due to the low dependence between the chemical and disease entities.

#### Performance comparison among C*_S_*, C*_D_* and their combination

In our experiments, the document-level classifier $C_{\text{D}}$ outperformed the sentence-level classifier $C_{\text{S}}$ in both the end-to-end setting and the gold-standard settings (see Table 4). One obvious reason is that the $C_{\text{S}}$ discarded the chemical-induced disease pairs across multiple sentences, which accounted for ∼30% of the CID relations in the corpus (24). Moreover, the automatically generated corpus for the $C_{\text{S}}$ approach was based on a simple assumption, and it contained many false positive instances. The combination of $C_{\text{D}}$ and $C_{\text{S}}$ achieved the highest *F*-score of 0.5853 and 0.6716 on the end-to-end setting and the gold-standard setting, respectively (Table 4). Regarding the performance of individual classifiers, the $C_{\text{S}}$ achieved an *F*-score of 0.5209 and the $C_{\text{D}}$ achieved an *F*-score of 0.5648, respectively. These *F*-scores were still among the top-ranked submissions in the BioCreative V CDR challenge.

#### The contribution of features from domain knowledge bases

The features derived from domain-specific knowledge bases improved the CDR extraction performance. As illustrated in Table 5, domain knowledge played a critical role in CDR extraction. The contribution from different knowledge bases varied. The features derived from the CTD yielded the most improvement, which is not surprising, as CTD is the database for chemical-induced diseases. We also noticed that the category-related features derived from the MeSH improved performance on the recall.

### Error analysis

For the NER and normalization task, the incorrectly recognized boundaries of mentions caused a significant performance drop, especially for disease entities. Our system achieved an *F*-score of over 0.90 on disease recognition under relaxed matching which allows for boundaries overlapping. Most of the boundary errors were caused by missing modifiers in disease mentions, such as course and severity. For example, our system detected “hepatic failure” instead of “end-stage hepatic failure,” “hepatitis” instead of “acute hepatitis” and “liver injury” instead of “drug-induced liver injury.” One limitation of our system is that we did not handle abbreviations well at this time. For example, in “indomethacin (IDM)”, although the long form mention “IDM” was correctly recognized, our system missed “IDM” as a chemical in following sentences. Errors caused by missing abbreviations occurred for both diseases and chemicals.

There are various types of errors for the CID task. First, implicit relations that are across multiple sentences are difficult to detect. Another type of error was related to disease granularities. For example, there was explicit evidence in the abstract that chemical X induced disease Y. However, in gold standard, a relation pair <X, Z> was extracted instead of <X, Y> in many cases, because Z was a more specific disease of Y. Moreover, the errors propagated from the NER and normalization step also reduced the performance of the end-to-end system. As we seen from Table 4, the performance of the system increased ∼10%, when the gold-standard entities were used.

## Conclusion

In this study, we incorporated machine-learning algorithms with domain-specific knowledge to build an end-to-end system for chemical-induced disease relation extraction, which consists of a disease and chemical NER and normalization module and a chemical-induced disease relation extraction module. In the BioCreative V CDR Track, our system achieved the highest performance on the CID task, indicating the feasibility of the proposed approaches for chemical-induced disease relation extraction.
